# Investigation of natural effective gamma dose rates case study: Ardebil Province in Iran

**DOI:** 10.1186/1735-2746-9-1

**Published:** 2012-08-02

**Authors:** Sadegh Hazrati, Abbas Naghizadeh Baghi, Hadi Sadeghi, Manouchehr Barak, Sahar Zivari, Soheila Rahimzadeh

**Affiliations:** 1 Department of Environmental Health, School of Public Health, Ardebil University of Medical Sciences, Ardebil, Iran; 2 Department of Basic Sciences, School of Medicine, Ardebil University of Medical Sciences, Ardebil, Iran; 3 School of Medicine, Ardebil University of Medical Sciences, Ardebil, Iran; 4 Ardebil Province Health Center, Ardebil University of Medical Sciences, Ardebil, Iran

**Keywords:** Background gamma, Ardebil, Outdoor, Dose rate

## Abstract

Gamma rays pose enough energy to induce chemical changes that may be biologically important for the normal functioning of body cells. The external exposure of human beings to natural environmental gamma radiation normally exceeds that from all man-made sources combined. In this research natural background gamma dose rates and corresponding annual effective doses were determined for selected cities of Ardebil province. Outdoor gamma dose rates were measured using an Ion Chamber Survey Meter in 105 locations in selected districts. Average absorbed doses for Ardebil, Sar-Ein, Germy, Neer, Shourabil Recreational Lake, and Kosar were determined as 265, 219, 344, 233, 352, and 358 nSv/h, respectively. Although dose rates recorded for Germi and Kosar are comparable with some areas with high natural radiation background, however, the dose rates in other districts are well below the levels reported for such locations. Average annual effective dose due to indoor and outdoor gamma radiation for Ardebil province was estimated as 1.73 (1.35–2.39) mSv, which is on average 2 times higher than the world population weighted average.

## Introduction

Natural ionizing radiation is emitted as a result of spontaneous nuclear transformations of unstable radionuclides naturally occurring in the earth’s crust (i.e. terrestrial origin) as well as those coming from outer space into the atmosphere (i.e. extraterrestrial origin). Gamma radiations as electromagnetic rays often accompany with emission of alpha or beta particles from a nucleus. The majority of human exposure to ionizing radiation occurs from natural sources including cosmic rays and terrestrial radiation [1]. Exposure to extraterrestrial origin radiation, galactic cosmic rays and energetic particles from solar particle events depends mostly on geographical characteristics of a place such as altitude, latitude, and solar activity [2,3]. The interaction of cosmic radiation with atoms in the atmosphere produce a range of radionuclides that can give rise to human exposure by inhalation or by ingestion after their uptake by plants [4].

Natural radionuclides of terrestrial origin have very long half-lives or driven from very long-lived parent radionuclides, which have been created in stellar processes before the earth formation. Naturally occurring primordial radionuclides mainly include ^238^U, ^235^U, and ^232^Th series and ^40^ K [5]. Unlike the pollutants with anthropogenic sources (e.g. polybrominated diphenyl ethers) that are introduced into environment through human activity [6], terrestrial origin radionuclides are naturally present at trace levels in all environmental compartments. Most radionuclides in the uranium and thorium series and ^40^ K emit gamma radiation, giving rise to exposures from gamma rays outdoor.

Gamma ray accounts for the majority of external human exposure to radiation from all source types due to its high penetration ability [7]. Gamma radiation has sufficient energy to eject one or more orbital electrons from atoms in the human body and hence break chemical bonds through non-thermal process, thus inducing chemical changes that may be biologically important for the normal functioning of body cells. Physical and chemical processes occurring following the radiation exposure involve successive changes at the molecular, cellular, tissue, and whole body levels that may lead to a wide range of health effects varying from simple irritation, radiation-induced cancer, and hereditary disorders to immediate death [2,8]. Unlike the electromagnetic fields that mostly limited to some specific locations [9], gamma radiation is ubiquitous. Great variations have been observed in environmental radiation levels and that several national and international studies have been characterized gamma dose rates both in outdoor and indoor environments [10-14]. [3,15] reports indicate that world population weighted values for external exposure from terrestrial gamma radiation in outdoors, indoors, and that from cosmic rays at sea level are 59, 84, and 30.9 nGy /h. High levels of environmental gamma radiation are expected in Ardebil province, in northwestern Iran, due to high altitude from the sea level and magmatic highlands (i.e. Sabalan Mountain) located in middle of the state. Since there was no comprehensive report on radiation exposure in this area, background gamma dose rates were measured in selected districts of Ardebil province.

## Materials and methods

### Selection of the measurement sites

**Figure 1:**
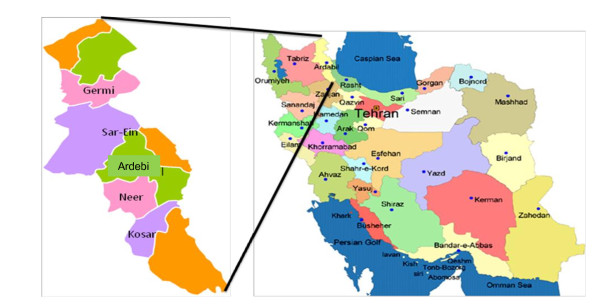
Locations of the selected cities (Germi, Sar-Ein, Ardebil, Neer, and Kosar) in north west of Iran.

**Figure 2:**
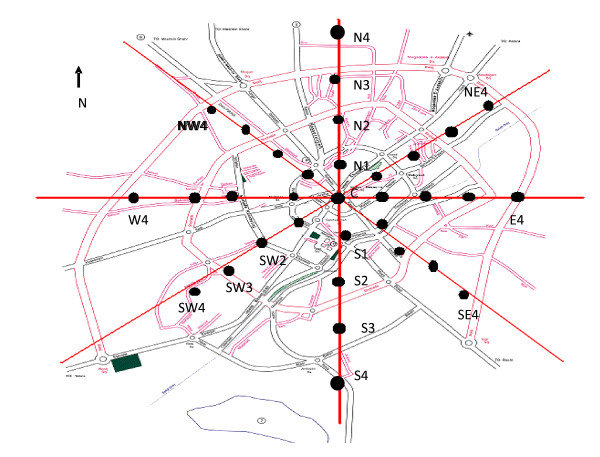
Selection of the measurement sites in Ardebil city.

Environmental gamma dose rates were measured in 5 out of 10 districts (i.e. Ardebil, Sar-Ein, Germy, Neer, and Kosar) within Ardebil province (Figure 1) as well as in Shorabil Recreational Lake located at south of Ardebil city from 2009 to 2010. These locations include about 66% of urban dwellers of Ardebil province [16]. For each district, the city center was assumed as a reference point and additional sites were selected in both cardinal and ordinal directions with an appropriate distance from each other (Figure 2). Based on the size of the districts; 33, 17, 21, 17, and 17 locations were monitored in Ardebil, Sar-Ein, Germy, Neer, and Kosar, respectively. 18 locations around Shorabil Recreational Lake were also monitored.

### Dose rate measurement

Environmental gamma dose rates were measured using an Ion Chamber Survey Meter, FLuke-451b, in 105 locations. The measurements were performed both at 20 and 100 cm above the ground for a period of one hour. A minimum distance of 6 m from buildings was kept for each measurement campaign [17]. A well designed stand was employed to obtain above-mentioned measuring heights. The instrument was calibrated in an Iranian Atomic Energy Agency accredited laboratory using ^137^Cs prior to gamma dose rate measurement and a calibration factor of 1 was obtained for the dosimeter. Slide of the dosimeter was kept closed during the measurement campaign in order to prevent the effect of other ionizing particles (i.e. alpha and beta) on recorded dose rates.

### Calculation of effective absorbed dose rate

Biological effects of ionizing radiation on human are evaluated based on the effective absorbed dose rate. Annual effective absorbed dose was determined using the algorithm below:

(1) $$HE_{\mathrm{total}}=HE_{\mathrm{Out}}+HE_{\mathrm{In}}\;$$

(2) $$HE_{\mathrm{out}}=T\times {}^{\circ}Dout\times C_{C}\times OF_{\mathrm{out}}\times 10^{-6}\;$$

(3) $$HE_{\mathrm{In}}=T\times {}^{\circ}D_{\mathrm{In}}\times C_{C}\times OF_{\mathrm{In}}\times 10^{-6}\;$$

Where:

HE_Total_ = Total annual effective absorbed dose rate (mSv/y)

HE_In_ = Indoor annual effective absorbed dose rate (mSv/y)

HE_Out_ = Outdoor annual effective absorbed dose rate (mSv/y)

T = Time in hours (8760 hours for a year)

*D*_*In*_=Absorbed dose rate in indoor (nSv/h)

*D*_*Out*_=Absorbed dose rate in outdoor (nSv/h)

C_C_ = Correction coefficient (0.7 for adult) [18]

OF_In/Out_ = Occupancy factor (80% for indoor and 20% for outdoor) [15]

## Results

The determined average absorbed dose rates at 20 and 100 cm above the ground for selected districts are summarized in Table 1. Gamma dose rate for both 20 and 100 cm did not significantly deviate from normal distribution (Komsgrove Sminov, p>0.05) and that, paired *T*-test was applied to compare dose rates at two different heights for all sites monitored. Statistical analysis showed that there are no significant differences in gamma dose rates at two heights (20 and 100 cm) for all locations except Kosar city. Therefore, average of dose rates at two heights was applied for further analysis.

**Table 1:** Average absorbed dose rates (μSv/h) at 20 and 100 cm above the ground for locations monitored

**Location**		**Average**	**SD**	**Min.**	**25**^**th**^**percentile**	**Median**	**75**^**th**^**percentile**	**Max.**
Shourabil Recreational Lake	20 cm	331	183	120	165	319	378	707
	100 cm	374	174	100	227	420	500	633
Ardebil	20 cm	285	125	133	200	260	333	647
	100 cm	246	106	120	187	220	267	700
Sar-Ein	20 cm	206	76	113	160	187	267	353
	100 cm	233	87	107	193	213	253	440
Germi	20 cm	331	112	130	260	360	390	640
	100 cm	357	136	140	290	360	440	720
Kosar	20 cm	322	39	260	290	320	330	410
	100 cm	394	82	260	340	390	460	520
Neer	20 cm	231	28	180	210	240	260	270
	100 cm	234	33	190	200	240	250	320

Average of doses measured for 20 and 100 cm heights along with estimated annual effective absorbed dose rates due to outdoor and indoor gamma rays are presented in Table 2. The highest dose rate was observed in Kosar and the lowest in Sar-Ein with respective values of 358 and 219 μSv/h.

**Table 2:** Estimated annual effective absorbed dose in selected districts of Ardebil province (mSv)

**Location**	**Average dose rate in outdoor (nSv/h)**	**Average dose rate in indoor (nSv/h)**	**HE**^**Out**^		**HE**^In^*	**HE**^**Total**^
			**Mean**	**SD**	**Mean**	
Ardebil	265	238*	0.325	0.124	1.17	1.495
Sar-Ein	219	221*	0.269	0.083	1.08	1.349
Germi	344	402*	0.422	0.142	1.97	2.392
Kosar	358	361*	0.439	0.069	1.77	2.209
Neer	233	164	0.285	0.028	0.804	1.089
Average	284	277	0.340		1.393	1.733

* Indoor annual effective absorbed dose rates have been taken from [25].

## Discussion

### Outdoor gamma dose rate

**Figure 3:**
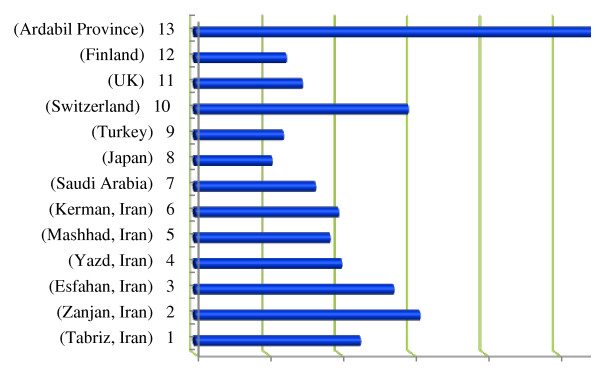
**Outdoor natural gamma dose rates (nGy/h) reported for some locations.** Average annual effective dose for Ardebil province was 202 estimated to be 1.73 mSv ranging from 1.35 (for Sar-Ein) 203 to 2.39 mSv (For Germi).

Average absorbed dose (i.e. arithmetic average of environmental gamma dose rate at 20 and 100 cm above the ground) for Ardebil, Sar-Ein, Germy, Neer, Shourabil Recreational Lake, and Kosar were 265, 219, 344, 233, 352, and 358 nSv/h, respectively. These values are due to both, terrestrial and cosmic radiation sources. Wide variation was observed in gamma dose rates quantified in different locations ranging from 110 to 680 nSv/h. Provided that altitudes of all districts studied are relatively similar; the variations observed in gamma dose rates might be attributed to differences in soil composition, since two cities with the highest dose rates (i.e. Germi and Kosar) are located on rocky areas. Gamma dose rates recorded for these locations are higher than those values reported for other cities within Iran (e.g. Tabriz, Zanjan, Esfahan, Yazd, Mashhad, and Kerman), (Figure 3) but still much lower than some areas (i.e. Ramsar, Mahallat and HezarMasjed) that have been classified as areas with high natural radiation background [15,19,20]. In Ramsar and Mahallat, surveys of outdoor radiation doses showed potential gamma exposures of 70–17000 and 800–4000 nGy/h, respectively [19]. Furthermore, a study carried out in high radiation areas of southwest coast of India showed an average dose arte of 200–4000 nGy/h [15,19]. Although dose rates recorded for Germi and Kosar are comparable with some areas with high natural radiation background (e.g.Yangjiang Quangdong in China, Campania in Italy, and Tessin in Switzerland) [15], however, the dose rates in other districts are well below the levels reported for such locations.

On the other hand, gamma dose rates reported for Switzerland, the UK, Saudi Arabia, Japan, and Turkey are lower than the rates measured in this study (Figure 3). United Nations Scientific Committee on the Effects of Atomic Radiation (UNSCEAR) estimated global dose rate due to cosmic rays and terrestrial gamma radiation to be on average 89 nGy/h. Assuming this dose rate as a normal level, doses quantified in Ardebil province are 2.4 to 4 times higher than worldwide population weighted average. The exact reason for high radiation doses are not known, but might be attributed to geographical, geological, and altitude of cities studied. In order to put in context, the results obtained in this study along with the values reported for some other locations are provided in Figure 3.

Data for 1 have been taken from [21], 2 from [13], 3 from [14], 4 from [17], 5 from [22], 6 from [23], 7 from [10], 8 from [15], 9 from [24], 10 from [12], 11 from [4], 12 from [11], and 13 represents the current study.

### Annual effective absorbed dose

Average annual effective dose for Ardebil province was estimated to be 1.73 mSv ranging from 1.35 (for Sar-Ein) to 2.39 mSv (For Germi). Based on the report of UNSCEAR, population weighed average of effective environmental gamma dose rate due to cosmic rays and terrestrial gamma radiation is 0.87 mGy/y. The annual effective environmental gamma dose rates due to indoor and outdoor for Ardebil province (Table 2) are appreciably higher than the values estimated for world average and that people living in Ardebil province receive on average 2 times (ranging from 1.3 to 2.7) higher environmental gamma radiation than the world population weighted average. Highest annual dose rates were observed in Kosar and Germi districts with respective values of 2.4 and 2.2 mSv.

## Competing interests

The authors declare that they have no competing interests. 

## Authors’ contributions

S. Hazrati developed initial idea and proposed and supervised the whole work. A. Naghizadeh Baghi administered data collection. H.Sadeghi supervised the research process in Germi City M. Barak as a health deputy of Ardabil University of Medical Sciences helped to get access to the monitoring places. S. Zivari involved in data collection and conduction of the work in Shorabil Recreational Lake S. Rahimzadeh involved in data collection and analysis in the work carried out in Ardabil and SarEin cities.
